# Addressing childhood trauma in a developmental context

**DOI:** 10.2989/17280583.2013.795154

**Published:** 2013-06-10

**Authors:** Claire Gregorowski, Soraya Seedat

**Affiliations:** Department of Psychiatry, Faculty of Health Sciences, Stellenbosch University

## Abstract

With the anticipated publication of the DSM-5 in May 2013, much reflection and work has been done on reviewing existing psychiatric nomenclature including, but not limited to the field of traumatic exposure. Traditionally, understanding of the psychiatric and psychological effects of trauma have been developed from studies with adults and then applied to trauma-exposed children with some modifications. While this is an important step to understanding the sequelae of trauma in children and adolescents, the adverse developmental effects of traumatic exposures on the rapidly evolving neurological, physical, social and psychological capacities of children calls for a developmentally sensitive framework for understanding, assessing and treating trauma-exposed children. The importance of early attachment relationships in infancy and childhood means that severely disrupted early caregiving relationships may have far-reaching and lifelong developmental consequences and can therefore be considered traumatic. Given the high rates of violence and trauma exposure of South African children and adolescents, the need for a developmentally based understanding of the effects of trauma on child and adolescent mental health becomes even more pronounced. In this paper, we draw on theoretical perspectives to provide a practical, clinically driven approach to the management of developmental trauma.

## Introduction

Developmental trauma refers to exposure to multiple, cumulative traumatic events, usually of an interpersonal nature, during childhood which results in developmentally adverse consequences (van der Kolk 2005, Busuttil 2009). It can include such experiences as sexual, physical, and emotional abuse, neglect, war, community violence, traumatic loss, betrayal or disruption of primary attachment relationships and chronic emotional dysregulation of caregivers (van der Kolk 2005, Ford and Cloitre 2009, Sarr 2011). Over the past 10 years, researchers have developed the concept of developmental trauma (van der Kolk 2005). This article highlights the theoretical concept of developmental trauma as a useful framework for the clinical understanding and management of South African children and adolescents with a history of maltreatment, multiple, prolonged trauma exposure or both. It includes a brief discussion of some research evidence supporting the concept, discusses the proposed DSM 5 diagnostic criteria for post-traumatic stress disorder in children and adolescents, and argues for the clinical utility of the concept of developmental trauma in the South African context.

## An overview of developmental trauma

The diagnostic category of Developmental Trauma Disorder (DTD) was conceptualised by the Complex Trauma taskforce of the National Child Traumatic Stress Network (NCTSN) (Pynoos *et al*. 2008). This category describes the subjective experience and effects of cumulative and developmentally adverse traumas in children and adolescents within the context of “significant disruptions of protective caregiving” (van der Kolk 2005, van der Kolk *et al*. 2009: 6). DTD comprises three main components. Firstly, the child or adolescent experiences multiple forms of persistent dysregulation in response to traumatic reminders. Dysregulation pertains to emotional, somatic, behavioural, cognitive and relational (including attachment) difficulties, as well as the development of dysfunctional attributions towards the self (van der Kolk 2005). Secondly, these triggers are often generalised to non-traumatic stimuli, leading to frequent and pervasive traumatic responses. Thirdly, behaviour becomes organised in anticipation of, and with attempts to, prevent recurrence of the traumatic experience (van der Kolk 2005). Children's or adolescents' attributions towards themselves, others and their environment are altered to reflect negative thoughts about themselves and their future, as well as expectations of future re-traumatisation and absence of care and protection from others. The following section considers early attachment experiences and the development and impact of various forms of dysregulation, in the context of early, chronic interpersonal trauma exposure.

### Attachment and emotional dysregulation

Dysregulation of affect can include emotional lability, numbing, difficulty identifying and verbalising affective states and poor communication of needs (van der Kolk 2005, Ford and Courtois 2009). The basis of our ability to function in the world stems from our early relational (attachment) experiences (Schore 1994, Cook *et al*. 2005). The mechanisms through which early relational experiences have an impact on emotional regulation and functioning have been described in detail in attachment theory (see Bowlby 1969, 1978), object relations theory (see Winnicott 1958, 1965), developmental theory (see Mahler, Pine and Bergman 1975), neurobiological theory (see Schore 1994, 2003a, 2003b), self-psychological theory (see Kohut 1971, 1977) and mentalisation theory (see Fonagy and Target 1997, Fonagy 2002, Fonagy, Gergely and Target 2007) among others.

When children experience disrupted and insecure attachment (i.e. through abuse, traumatic loss, betrayal, or as a result of chronic dysregulation in the caregiver), they are unable to develop the capacity to self-regulate, and therefore experience intense feelings without the ability to modulate these themselves, or to rely on safe and consistent caregiving relationships for support (Cook *et al*. 2005, van der Kolk 2005). Chronically traumatised children therefore struggle to identify and communicate their feelings and lack a sense of agency regarding their own internal experience and their ability to influence the world around them (Cook *et al*. 2005). The resulting helplessness can lead to excessive clinginess, excessive anxiety, internally or externally directed aggression and dissociation (Cook *et al*. 2005, van der Kolk 2005, Bailey, Moran and Pederson 2007).

### Cognition and learning

Developmental delay is often associated with childhood maltreatment, leading to potential impaired cognitive functioning and learning difficulties (Cook *et al*. 2005, van der Kolk 2005). Research suggests that the need for chronically traumatised children to focus on survival through avoiding or being over-reliant on caregiver relationships and becoming hyper-vigilant towards potential threats leads to structural changes in brain development, impacting later on attention, learning and memory capacities (Ford 2005). During the first two years, children develop important cognitive capacities such as symbolism, the beginnings of language and autobiographical self-awareness (i.e. self which is aware of being a subject who has knowledge and can act upon the world) (Ford 2005). These capacities develop further during the third and fourth years to include consistent schemas of the self and others over time, the ability to differentiate between emotions/intentions and impulses/actions, and the capacity to anticipate future occurrences by integrating new information with past experience (Cook *et al.* 2005, Ford 2005). Chronic traumatisation during these developmental stages may lead to the faulty categorisation of information where almost all stimuli and experiences are interpreted as potentially traumatic and responded to accordingly (van der Kolk and Courtois 2005). Curiosity is therefore restricted and learning inhibited. This may result in over-developed memory for traumatic events and deficits in “attention, hypothesis testing, problem solving, semantic organisation and short-term and delayed semantic memory” (Ford 2005: 417). The cognitive capacities necessary for an integrated, continuous sense of self may also be impaired.

### Consciousness

According to Ford and Cloitre (2009), ongoing avoidance and hyper-vigilance in the face of chronic traumatisation can become automatic rather than conscious, leading to dissociation and fragmented consciousness. Dissociation can also develop as a psychic defence against the anxiety associated with recognising a child's own helplessness or the caregiver's potential danger (Bailey *et al*. 2007). According to Cook *et al*. (2005), chronically traumatised children may use dissociation adaptively in three important ways: firstly through responding automatically without appropriate judgment, planning or organisation; secondly, through isolating painful affects and memories; and thirdly, through disconnecting from awareness of feelings and the self. However, the use of such defences prevents the integration of other memories and experiences, leading to a poorly integrated sense of self over time and a distorted sense of self and others (Cook *et al*. 2005, Bailey *et al*. 2007, Fonagy *et al*. 2007, Ford and Cloitre 2009). Further maladaptive consequences include disconnection between thoughts and feelings, lack of conscious awareness of bodily sensations and behavioural re-enactments outside of awareness or control (Cook *et al*. 2005, Ford and Cloitre 2009). Difficulties in emotional awareness may combine with affect dysregulation to have a negative impact on attachment relationships (Cook *et al*. 2005).

### Behavioural difficulties

Deficits in the ability to modulate affect results in a lack of impulse control (Cook *et al*. 2005, van der Kolk 2005, Bailey *et al*. 2007). The experience of intense affect such as rage and shame and the associated difficulties with affective regulation can also lead to children engaging in withdrawal or behavioural enactments. These are aimed at avoiding intense emotional states, generating a subjective sense of control or mastery to protect themselves from the recurrence of these terrifying feelings, or attempting to gain contact or intimacy with another (Cook *et al*. 2005, van der Kolk 2005). Children also tend to communicate their traumatic experiences through behavioural enactments where they either take the part of the abuser or the abused, for example becoming aggressive, engaging in deliberate self-harm, exhibiting sexualised behaviour or trying to control relationships (Cook *et al*. 2005, van der Kolk 2005). Behavioural difficulties such as social withdrawal, over compliance, impulsivity, aggression and defiance place additional strain on attachment relationships and may prevent the development of potentially supportive, mitigating relationships during childhood and adolescence, thus compounding the problem. Insecure attachment is also associated with later maladaptive coping behaviours such as substance abuse, eating disorders or high risk behaviours (Ford 2005).

### Somatic/biological distress

The experience of chronic trauma can lead to physical discomfort and distress through several mechanisms. Physical abuse and neglect can lead directly to physical injuries or illnesses. Traumatic stress reactions such as physiological dysregulation can cause or exacerbate medical conditions, including orthopaedic, neurological and cardiovascular illnesses (Cook *et al*. 2005, Ford and Cloitre 2009). Somatic complaints such as headaches and stomach aches may develop as a response to intense feelings in the absence of better modes of communication and these can intensify to include conversion symptoms or forms of dissociation such as pseudo-seizures (Cook *et al*. 2005, Ford and Cloitre 2009).

### Altered attributions towards the self and others

Significant consequences of dysregulation and disrupted attachment include a poor self-concept, a sense of the world as dangerous and help as unavailable or futile (van der Kolk, 2005). Repeated experiences of rejection, betrayal and abuse lead to children feeling they are bad, worthless or not worthy of being loved. This combined with poor developmental competencies and subsequent feelings of ineptitude lead to self-blame and guilt (Cook *et al*. 2005). Negative self—and other—attributions and expectations further exacerbate the already described tendency towards hyper-vigilance and faulty information processing. Responses to both neutral and traumatic stimuli tend to be confused and disorganised, leading to further self-perceptions of helplessness (van der Kolk 2005).

Dysregulation and negative attributions may result in functional difficulties in all areas such as familial and social relationships, educational and vocational achievement, even leading to legal difficulties (van der Kolk 2005). Developmental trauma is considered the aetiological basis of Complex Post-traumatic Stress Disorder (CPTSD) in adulthood (Bailey *et al*. 2007, Sarr 2011).

## Research evidence

The lack of a developmentally appropriate psychiatric nosology for the pervasive emotional, behavioural and neurobiological effects of developmental trauma means that children and adults with a history of developmental trauma are often diagnosed with comorbid psychiatric and medical disorders where the common aetiological factor of chronic trauma exposure can go unrecognised (van der Kolk *et al*. 2009, Lieberman *et al*. 2011, Schmid, Petermann and Fegert 2013).

Research has highlighted the intensity, diversity and complexity of symptomatology following developmental trauma (Cloitre *et al*. 2009, Greeson *et al*. 2009, Benjet, Borges and Medina-Mora 2010, Cutajar *et al*. 2010, Lieberman *et al*. 2011). Ford (2011) found that a history of exposure to interpersonal violence in children was associated with increased severity of disruptive behaviour problems compared with non-interpersonal violence exposure, including when controlling for psychiatric diagnoses and demographics. Dorsey *et al*. (2012) found an association between childhood abuse, domestic violence, neglect and death or incarceration of a parent or caregiver and placement in treatment foster care.

The mediating role of emotional dysregulation in the development of mental health difficulties for children with a history of abuse or maltreatment has been highlighted in recent literature (Wanner *et al*. 2012). Furthermore, increasing evidence is available for the association between childhood maltreatment, disrupted attachment and emotional and behavioural difficulties in children and adolescents (Kira *et al*. 2012, Joubert, Webster and Hackett 2012). Studies also indicate that cumulative childhood trauma may lead to the development of psychiatric disorders in childhood and/or adulthood (see Table 1).

**Table 1.** Psychiatric sequelae of cumulative childhood trauma

Psychiatric sequelae	Source
Mood disorders	Didie *et al*. 2006, Benjet *et al*. 2010, Shenk *et al*. 2010, Collin-Vézina *et al*. 2011 Lieberman *et al*. 2011
Attempted suicide	Didie *et al*. 2006, Lieberman *et al*. 2011, Wanner *et al*. 2012
Self-injurious behaviour	McReynolds and Wasserman 2011
Anxiety disorders	Greeson *et al*. 2009, Benjet *et al*. 2010, Cougle *et al*. 2010, Cutajar *et al*. 2010, Collin-Vézina *et al.* 2011
Externalising disorders (intermittent explosive disorder, oppositional defiant disorder, conduct disorder and ADHD)	Benjet *et al*. 2010, Shenk *et al*. 2010, Muller *et al*. 2013
Aggression	Silvern and Griese 2012
Substance use disorders	Benjet *et al*. 2010, Cutajar *et al*. 2010, Tucci, Kerr-Corrêa and Souza-Formigon 2010, Lieberman *et al*. 2011
Psychosis	Cutajar *et al*. 2010
Personality disorders (most commonly borderline personality disorder)	Berenbaum 1996, Cutajar *et al*. 2010
Alexithymia	Berenbaum 1996
Dissociation	Bailey *et al*. 2007, Collin-Vézina *et al*. 2011, Silvern and Griese 2012
Catatonia	Dhossche, Ross and Stoppelbein 2012
Somatisation	Nickel and Egle 2006
Eating disorders	Jaite *et al*. 2011
Body dysmorphic disorder	Didie *et al*. 2006
Cognitive difficulties	Majer *et al*. 2010, Kira *et al*. 2012, Spann *et al*. 2012

Van der Kolk *et al*. (2009) have developed consensus proposed diagnostic criteria for DTD. They argue for the inclusion of DTD in DSM 5, citing various studies which provide evidence for the validity and reliability of this diagnosis. This issue has generated some debate. D'Andrea *et al*. (2012) provide a review of current literature evidencing responses to early, chronic interpersonal trauma, including an outline of the biological correlates of symptoms observed in maltreated children. They conclude that sufficient evidence exists to argue for DTD as a distinct diagnostic category from PTSD, or at very least a different framework warranting further research. However, Resick *et al*. (2012) and Schmid *et al*. (2013) in their reviews of the literature on CPTSD and DTD respectively, conclude that while these concepts have usefully highlighted the role of chronic early trauma in the development of complex psychopathology in childhood and adulthood, more research (especially longitudinal) is needed before CPTSD or DTD should be recognised as separate diagnostic classifications. Lindauer (2012), however, argues that the complicated nature of complex trauma makes it difficult to evidence clear causal relationships, and suggests that the clinical utility of the concept and its ability to increase the awareness and structural responsiveness to children and adolescents experiencing the sequelae of developmental trauma should not be sacrificed to scientific principles that may be overly strict. The following section outlines some of the current literature on the place of child and adolescent responses to trauma in DSM 5.

## Developmental trauma and DSM-5

Currently, trauma exposed children are psychiatrically assessed and diagnosed according to adult PTSD diagnostic criteria of the Diagnostic and Statistical Manual for Mental Disorders, 4th edition, text-revision (DSM-IV-TR) (APA 2000, Scheeringa, Zeanah and Cohen 2011). However, researchers and clinicians have for some time raised concerns that these diagnostic criteria (which were developed based on data for patients aged 15 years and older) do not adequately take into account developmental considerations, leading to an under-diagnosis of the disorder in highly symptomatic young children (van der Kolk 2005, Pynoos *et al*. 2009, van der Kolk *et al*. 2009, De Young, Kenardy and Cobham 2011, Scheeringa *et al*. 2011, 2012, Blom and Overink 2012).

Scheeringa *et al*. (2012) propose an alternative, developmentally sensitive set of diagnostic criteria for PTSD in preschool children (aged 6 years and younger), which they label an alternative algorithm (PTSD-AA). There are several differences between the DSM-IV-TR PTSD diagnostic criteria and PTSD-AA. First is the removal of criterion A2 (an initial response of fear, helplessness or horror). This criterion is considered unreliable in young children because of limited verbal expression and the possibility that caregivers may not be present to witness such responses. The inclusion of this criterion may therefore lead to under-diagnosis of otherwise symptomatic children. These authors argue that even though the DSM-IV-TR provides provision for the behavioural manifestation of ‘disorganised or agitated’ behaviour in children, these descriptors are unclear and empirical evidence on their validity is lacking (Scheeringa *et al*. 2011, 2012). Secondly, a note has been added to criterion B1 that not all children exhibit distress; this was done to take into account research that suggests that some children exhibit “overbright positive emotional displays” (Scheeringa *et al*. 2011: 773) in response to intrusive traumatic reminders (Scheeringa *et al*. 2011). Thirdly, the PTSD-AA diagnostic criteria require only one avoidance or numbing response (criteria C1-7). The authors argue that these symptoms are often internalised, and children are developmentally unable to verbally identify and express such internal states. As a result, only four out of the seven criteria symptoms could reliably be expressed by children or noticed by adults, making under-diagnosis more likely (Scheeringa *et al*. 2011, 2012). Qualification has also been made to some of the wording of criteria, for example, criterion 4 states that ‘diminished interest in significant activities’ may present as restricted play. Some criteria have substituted more developmentally appropriate wording, such as ‘detachment from others’ being replaced by ‘socially withdrawn behaviour’ (criterion C5). Finally, ‘extreme temper tantrums’ has been added as a possible indication of irritability and anger (criterion D2) (Scheeringa *et al*. 2011, 2012). Researchers and clinicians have also raised concerns regarding criterion A1, arguing that some forms of interpersonal trauma (such as emotional abuse, neglect, separation from a caregiver or traumatic loss) may not qualify as a ‘traumatic event’ and yet are experienced by children as potentially life threatening because of their increased dependence on caregivers and adults for safety and survival (van der Kolk 2005, Pynoos *et al*. 2009, De Young *et al*. 2011, Scheeringa *et al*. 2011).

To consider these concerns, two of the DSM 5 work groups commissioned a review of the evidence considering the impact of developmental issues on PTSD in children and adolescents (Scheeringa *et al*. 2011). The review focused on evidence pertaining to symptomatic presentation of 2 age groups following traumatic exposure: preschool children and children aged 7–14 years (Scheeringa *et al*. 2011). The preliminary recommendations of this task force include a developmental subtype of PTSD for preschool children (PTSD-DSM-5-P) to be included in the Disorders Usually First Diagnosed in Infancy, Childhood and Adolescence section. This was based on the PTSD-AA and on two additional symptom criteria based on self related and defensive subjective reactions which remain under consideration, namely ‘substantially increased frequency of negative emotional states, for example, fear, guilt, sadness, shame, or confusion’ as part of the avoidance/numbing criteria, and ‘reckless or self-endangering behaviour’ as a symptom of increased arousal (Ford and Courtois 2009, De Young *et al*. 2011, Scheeringa *et al*. 2011, Scheeringa *et al*. 2012). Blom and Overink (2012) in their review of the literature recommend that the alternative algorithm of Scheeringa *et al*. (2011) should be used to diagnose PTSD in preschool children.

In relation to children aged 7–14 years, the task force recommended the expansion of criterion A1 to include early, chronic interpersonal trauma (such as loss, injury or death of a caregiver or experience of multiple traumatisation). This amendment would consider that because of children's dependence on adults for physical and psychological well-being, loss of a caregiver may be experienced as the most traumatising experience (even if the caregiver was the perpetrator of abuse) (Scheeringa *et al*. 2011). The task force also recommended that the timeframe indicating that symptoms are not present before the trauma be removed because the psychological sequelae of early developmental trauma may be experienced by the child as part of their personality rather than linked to the traumatic experience. Similarly to preschool children, the task force recommended removing criterion A2 (children may be reluctant to describe how they feel due to trust difficulties resulting from traumatic relational disruptions), or expanding it to include additional responses observed in children such as numbness, confusion, sadness or feeling frozen (Scheeringa *et al*. 2011). Blom and Overink (2012) similarly recommend expanding criterion A2 and note that while an association has been shown in the literature between this criterion and PTSD, youth can also be PTSD positive in the absence of this criterion. Scheeringa *et al*. (2011) report similar developmental concerns as with preschool children regarding the applicability of symptoms of criteria B, C and D to children aged 7–14 years, although they qualify that less empirical research is available for this population compared with preschool children (Scheeringa *et al*. 2011). Scheeringa *et al*. (2012) and De Young *et al*. (2011) reported improved validity of the proposed DSM-5 diagnostic criteria compared with the DSM-IV criteria in symptomatic, trauma exposed children.

Proposed changes to the DSM-5 may therefore improve diagnostic validity and ensure that more young children who have been exposed to developmental trauma receive treatment. However, as previously highlighted, PTSD symptoms may represent only part of the biopsychosocial sequelae of chronic, early trauma exposure. An argument can therefore be made for mental health professionals to extend assessment beyond even the proposed DSM 5 diagnostic algorithms to incorporate the assessment guidelines outlined below.

## Assessment and treatment

Assessment and treatment of children who have experienced multiple traumas must address symptoms, dysregulation and impairment in all affected domains of functioning: emotional, behavioural, cognitive, somatic and relational (Briere and Spinazzola 2005, Cook *et al*. 2005, Ford and Cloitre 2009). Assessment should include holistic evaluation of trauma exposure and post-traumatic sequelae, the child's personal and family history, attachment relationships and environment (including a history of involvement of other professionals or agencies), individual and family strengths, and protective factors (Cook *et al*. 2005). Modes of assessment should include information from the child and collateral sources of information, clinician observation and standardised scales (Cook *et al*. 2005). Some specific assessment measures have been developed to measure complex trauma in children (see Table 2).

**Table 2.** Specific Models for the Assessment and Treatment of Developmental Trauma

Model	Description	Source
**Assessment**		
Child Behaviour Checklist (CBCL)	Measures psychological distress in children following trauma and abuse.	Achenbach (2002 cited in Briere and Spinazzola 2005)
Trauma Symptom Checklist for Children (TSCC)	A self-report questionnaire assessing trauma symptoms in children ages 8–16 years.	Briere (1996 cited in Briere and Spinazzola 2005)
Child Sexual Behaviour Inventory (CSBI)	Assesses observed sexual behaviour over the previous six months in 2–12 year old children.	Friedrich (1998 cited in Briere and Spinazzola 2005)

**Treatment**		
Trauma Focused Cognitive Behavioural Therapy (TF-CBT)	Provides psycho-education; addresses parenting skills; teaches affect modulation and expression as well as relaxation techniques and coping skills; assists with narrative processing of the trauma; uses desensitisation techniques to reduce traumatic reactions to trauma reminders; and aims to improve the safety of the child.	Cohen (2005), Lieberman (2011)
Child-Parent Psychotherapy (CPP)	Combines psychoanalytic attachment and trauma theory with social learning and CBT techniques to start to restore emotional regulation and a positive attachment relationship to the caregiver and to enable the re-telling of the trauma in an integrated life narrative.	Lieberman and van Horn (2005, 2008) both cited in Lieberman (2011)
Trauma Systems Therapy (TST)	Targets the development of self-regulatory capacities in children and reduction of stress and risk in the child's environment.	Saxe *et al*. (2005)
Attachment, Self-Regulation and Competency (SRC) model	Focuses on the development of skills to overcome trauma related barriers to healthy development.	Kinniburgh *et al*. (2005)
Skills Training in Affect and Interpersonal Regulation (STAIR)	A group treatment model for adolescents which directly targets affect regulation and interpersonal difficulties before progressing to emotional processing of the trauma through the use of prolonged exposure techniques.	Cloitre *et al*. (2002), Cook *et al*. (2005)
Structured Psychotherapy for Adolescents Responding to Chronic Stress (SPARCS)	A group treatment model which aims to foster and augment current coping skills through validation and connection. It aims to address difficulties with emotional regulation, physical health, attention and information processing, self-perception and sense of meaning.	Cook *et al*. (2005), De Rosa *et al*. (2006)
Trauma Adaptive Recovery Group Education and Training (TARGET)	Teaches adolescents skills for affect and physical self-regulation, information processing, relational problem solving and coping with stress through the use of experiential exercises.	Cook *et al*. (2005), Ford and Russo (2006)

Treatment guidelines for children exposed to developmentally adverse and/or multiple, chronic traumatisation have been developed by the Complex Trauma Work Group of the NCTSN (Ford and Cloitre 2009). Complex trauma interventions require six components: (i) establishing safety; (ii) self-regulation; (iii) self-reflective information processing; (iv) integration of traumatic experience into the life narrative; (v) reengagement with relationships; and (vi) enhancement of positive affect (Cook *et al*. 2005). Treatment plans should be organised around ameliorating distress while simultaneously incorporating the child and caregiver's wishes and goals, and should draw on individual and family strengths, resiliencies and resources (Ford and Cloitre 2009). For specific treatment models, based on the above treatment principles along with evidence based treatments of PTSD, see Table 2. The following sections outline the recommended guidelines for treating children and adolescents experiencing developmental trauma.

### Safety

Establishing environmental safety and stability is necessarily the first requirement for treating traumatised children (Cook *et al*. 2005, Ford and Cloitre 2009). Many of the symptoms have developed as survival adaptations and ameliorating or removing such defensive adaptations in the context of ongoing trauma may be further detrimental to the child or adolescent. Utilising external services such as social welfare agencies may be necessary (Ford and Cloitre 2009). Safety is needed not only externally, but internally, meaning that deliberate self-harm and suicidal ideation needs to be addressed and the child and adolescent needs to (re)gain regulatory capacities (Ford and Cloitre 2009). They need to feel in control of rather than subject to their bodies, feelings and thoughts (Ford and Cloitre 2009). As in all forms of psychological treatment, safety in the therapeutic relationship is key.

### Self-regulation

The ability to modulate arousal levels and regain homeostasis following dysregulation is a fundamental goal which should inform all phases of treatment (Cook *et al*. 2005, Ford and Cloitre 2009). This will enable the child to begin to live rather than merely survive (Ford and Cloitre 2009). Appropriate recognition, labelling and expression of intense affect and bodily sensations can lead to improved impulse control and reduction in behavioural, somatic and relational difficulties because it enables children to use their internal experience to guide behaviour and develop informed, chosen responses (Ford and Cloitre 2009). In the case of bodily dysregulation, medical professionals may need to be consulted as pharmacotherapy may be needed alongside the focus on emotional regulatory capacities (Ford and Cloitre 2009). Helping a child to develop capacities to regulate affect requires providing them with opportunities to play, explore and learn. This should happen in parallel with the development of trusting relationships with consistent, reliable adults who can model how to recognise and name feelings, wishes and goals (Ford and Cloitre 2009). Group therapeutic interventions should be considered, especially in the case of older children and adolescents (Ford and Cloitre 2009).

### Self-reflective information processing

Addressing difficulties in attention, memory and executive functioning may better enable children to reflect on past and present experience and improve planning, problem solving and decision-making skills (Cook *et al*. 2005, Ford and Cloitre 2009). Techniques aimed at cognitive restructuring, development of mindfulness and narrative memory integration have been used (Ford and Cloitre 2009). In some cases treatment may require specialised neurocognitive rehabilitation, academic learning support or pharmacological intervention (Ford and Cloitre 2009). Dissociative experiences can also be addressed through modelling a mindful, reflective, step-by-step approach to planning daily tasks and activities, in a way which is cognizant of the child's emotional state (Ford and Cloitre 2009).

### Integration of traumatic experience

This requires the processing and integration of traumatic (and other) memories, and psychological and somatic sequelae of exposure to developmental trauma, into the child or adolescent's life narrative in such a way that they gain mastery over their traumatic memories (Cook *et al*. 2005, Ford and Cloitre 2009). Depending on the nature of the traumatic experience, current environment and level of functioning of the child, this may include various steps. These include (i) helping the child and caregiver recognise and anticipate traumatic responses; (ii) developing alternative coping strategies; (iii) reframing post-traumatic dysregulation as a previously adaptive response; (iv) helping the child mindfully alter these responses to become more adaptive; and (v) supporting the recall and storytelling of traumatic memories (Ford and Cloitre 2009).

### Re-engagement with relationships

(Re)gaining relationships with caregivers, supportive adults and peers needs to happen simultaneously with developing self-regulatory capacities in what Ford and Cloitre (2009: 73) term a “scaffolding” process. The experience of developmental trauma means that the child is likely to have experienced disrupted relationships, involving experiences from loss and betrayal to actual physical harm. The resulting negative expectations of the intentions and actions of others make it likely that even positive interactions will be experienced as potentially ineffectual or harmful, and may elicit traumatic memories and/or act as traumatic triggers for dysregulation (Ford and Cloitre 2009). Treatment involves helping children, caregivers and other supportive adults to anticipate, understand and cope with potential dysregulation (Ford and Cloitre 2009). Wherever possible, potential relational disruptions should be minimised, and well planned for and supported (Ford and Cloitre 2009). Therapists should also model and teach relational skills such as “assertiveness, cooperation, perspective-taking, boundaries and limit setting, reciprocity, social empathy, and the capacity for physical and emotional intimacy” (Cook *et al*. 2005: 394).

### Enhancement of positive affect

As children start to develop regulatory and relational competence, the experience of fun, mastery, creativity and pro-social activities should especially be facilitated (Cook *et al*. 2005). Curiosity and exploration should be encouraged and supported to facilitate learning (van der Kolk 2005). This will enable the development of positive self-esteem and self-evaluation (Cook *et al*. 2005).

## Application to the South African context

Developmental trauma may be a particularly useful framework for the management of traumatised children and adolescents in South Africa. The prevalence of child abuse in South Africa is high (Dawes and Ward 2011). Between March 2011 and April 2012, there were 54, 000 reported crimes against children in South Africa (Pawelczyk 2012). This figure is thought to be a significant underestimate as it does not include cases reported to non-governmental or other organisations and many cases of child maltreatment remain unreported (Pierce and Bozalek 2004). South African children and adolescents are also exposed to high levels of trauma and violence with figures of trauma exposure of between 40% and 100% being reported (Suliman, Kaminer and Seedat 2005). In a sample of 4 391 youth between the ages of 12 and 22 years, Leoschut (2009) reported that 41% had experienced at least one crime, 14.4% had experienced assault and 3.6% had experienced rape or sexual assault in their lifetime. Violence has also been reported between peers. According to Leoschut and Burton (2009), youth between the ages of 12 and 21 years are most likely to be the victims or perpetrators of violent crime. A study by Flisher *et al*. (2007) also reported that in a sample of 596 adolescents, 20.7% reported having perpetrated violence against their partner, and 16.4% reported an intention to do so.

There is, however, a paucity of epidemiological studies on the prevalence and impact of mental health difficulties of children and adolescents in South Africa (Flisher *et al*. 2012). Kleintjies *et al*. (2006) developed prevalence estimates for mental health disorders in children and adolescents in the Western Cape based on a review of the literature. They reported an overall estimated prevalence rate of 17%, with generalised anxiety disorder found to be most common (11%) followed by PTSD, and major depressive disorder/dysthymia (8% each), oppositional defiant disorder (6%), social phobia, enuresis and ADHD (5% each), separation anxiety disorder and conduct disorder (4% each), simple phobia and agoraphobia (3% each), bi-polar disorder (1%) and schizophrenia (0.5%). In an audit of patients seen by clinical psychologists working in state hospitals in Kokstad over a one year period, Pillay, Kometsi and Siyothula (2009) reported that of the 106 patients seen, 54.7% were children or adolescents (an over-representation compared with population rates). Of the patients referred for learning problems (31%), 90.9% were under 21 years, all of those referred for PTSD (*n* = 8) (of whom 5 were subsequently diagnosed with PTSD) were children or adolescents and of the children and adolescents, 82.8% were from “non-intact families” (Pillay *et al*. 2009: 292).

Studies have found associations between violence exposure and mental health difficulties such as clinical or subclinical PTSD (Flisher *et al*. 2001, Seedat 2005), depression and anxiety (Flisher *et al*. 2001) and symptoms of clinical distress including suicidal ideation and increased risk for psychiatric illness (Govender and Killian 2001). Exposure to other types of traumatic events such as abuse, neglect, loss, relational problems and psychosocial stressors has also been linked to mental health difficulties in the South African population. In a group of 669 children and adolescents referred for evaluation and treatment to the Child Mental Health Unit of the Free State Psychiatric Complex, 64.1% had experienced social stressors (including domestic violence, family substance abuse, financial problems, lack of support, parents’ divorce, problematic relations in the family, problems at school, and xenophobic attacks) and 19% experienced psychological stressors (such as emotional abuse, physical abuse, sexual abuse, hopelessness, isolation, low self-esteem, rape, rejection, worthlessness, nightmares, bereavement and behavioural problems). Of the same sample, 30% were diagnosed with attention deficit and disruptive behaviour disorders, 22.7% with major depressive disorders, 18.5% with anxiety disorders, 16.1% with conduct disorders, 9.6% with adjustment disorders, 8.8% with elimination disorders, and 7% with bereavement (Calitz *et al*. 2012). Carey *et al*. (2008) found a significant association between childhood sexual assault (CSA) and depression in a group of 40 girls. Other psychiatric diagnoses identified in this sample of children (*n* = 50) with a history of CSA included PTSD, adjustment disorder with depressed mood, panic disorder and separation anxiety disorder (Carey *et al*. 2008). Other mental health outcomes reported in the South African literature include PTSD (Traut *et al*. 2002, Suliman *et al*. 2005, 2009) and mood disorders (Suliman *et al*. 2009).

Flisher *et al*. (2012), in their consideration of the mental health service needs of children and adolescents in South Africa conclude that despite high levels of violence exposure and associated mental health difficulties, services for South African children and adolescents are often underdeveloped or inaccessible. Lund *et al*. (2009) report that current services in South Africa fall short of estimated minimal requirements. A cohesive, aetiologically based treatment approach to the complex and often comorbid psychological and psychiatric presentations of chronically traumatised children may help to ease this burden.

## Conclusion

The concept of developmental trauma has clinical utility in South Africa. The high levels of violence and child maltreatment mean that the mental health difficulties of children and adolescents are likely to be a result of or otherwise complicated by ongoing trauma exposure. Given the established links between childhood abuse, multiple, chronic traumatisation during childhood and childhood psychopathology, it is essential that when children come into contact with mental health services, the presence and aetiological salience of the experience of trauma (including developmental trauma) is assessed to inform both diagnosis and treatment. If not, treatment plans run the risk of being disjointed and inefficient at best, and ineffectual or re-traumatising at worst.

In a resource poor country such as South Africa, where minimal service requirements are not being met, the challenge is to develop efficient, cost-effective treatment for complex psychiatric presentations. Understanding developmental trauma as a common aetiological factor is a helpful start. Towards this end, self-report questionnaires could be used as screening tools for identifying children exposed to trauma. Professionals who have daily contact with children and adolescents should be educated regarding the effects of ongoing trauma exposure to ensure referrals are made to appropriate services. Further, there should be multi-disciplinary collaboration to ensure the child's internal and external safety. Treatment plans may need to involve the child's caregivers to improve attachment relationships and to make improvements gained in therapy more sustainable. Following initial treatment, support may need to be offered at critical periods during a child or adolescent's development when difficulties are likely to resurface. Group therapeutic processes such as those based on narrative therapy techniques could also be provided for children and adolescents, who are from communities identified as having high levels of violence and traumatisation, to promote healing and empowerment and improve mental health (Morkel 2011).
